# Relationship between serum cystatin C and polycystic ovary syndrome

**Published:** 2013-01

**Authors:** Mohammad Hossein Gozashti, Ahmad Gholamhosseinian, Fatemeh Musavi, Mahdieh Mashrouteh

**Affiliations:** 1 *Department of Endocrinology, Metabolism and Physiology Research Center, Kerman University of Medical Sciences, Kerman, Iran.*; 2 *Department of Biochemistry, Physiology Research Center, Kerman University of Medical Sciences, Kerman, Iran.*; 3 *Kerman University of Medical Sciences, Kerman, Iran.*

**Keywords:** *Polycystic ovary syndrome*, *Cystatin C*, *C-reactive protein*, *Dyslipidemia*, *Blood pressure*

## Abstract

**Background: **Polycystic ovary syndrome (PCOS) causes an increased risk of metabolic cardiovascular syndrome. Also, cystatin C serum levels are associated with the risk of cardiovascular events in metabolic syndrome patients.

**Objective:** To investigate the relationship between cystatin C in PCOS patients.

**Materials and Methods:** 35 women with PCOS were compared to 35 women with healthy matched age and body mass index. They all underwent tests to determine plasma levels of C-reactive protein (CRP), cystatin C, lipid profile and apo-lipoprotein. Blood pressure and demographic variables of each subject were obtained.

**Results:** Systolic and diastolic blood pressure were higher in PCOS patients compared to control group. Triglyceride and low-density lipoprotein cholesterol levels were higher in PCOS; contrariwise, high-density lipoprotein was lower from that of healthy volunteers. Cystatin and CRP levels were significantly higher in patients with PCOS in comparison with healthy subjects (p<0.0001). Among measured determinants, only PCOS status was independently associated with cystatin C.

**Conclusion:** Cystatin C was positively correlated with PCOS status concentrations but not with systolic and diastolic blood pressure, or any of the lipid profile variables or demographic characteristics. Indeed, no correlation was found between cystatin C and CRP levels. Therefore, cystatin C might be related to PCOS beyond its use as a marker of the renal function.

## Introduction

Polycystic ovary syndrome (PCOS) is a condition that frequently manifests during adolescence period affecting 5-10% of women of reproductive age and is the most common cause of menstrual irregularity and hirsutism (1). In 2003, the European Society for Human Reproduction and Embryology (ESHRE) and the American Society for Reproductive Medicine (ASRM) revised the definition of PCOS; the syndrome is now defined with the presence of two of the following three criteria: polycystic ovaries, oligo/anovulation and/or clinical or biochemical evidence of hyperandrogenism (2). 

The cause of PCOS is uncertain, but there is evidence for a primary abnormality of ovarian androgen production that is manifested at puberty but with origins that may very well root in childhood or even during fetal development (3). It is associated with chronic anovulation, insulin resistance, and androgen excess and is considered as one of the most common endocrine disorders among women of reproductive age (4, 5).

PCOS has been linked to an increased risk of metabolic cardiovascular syndrome (MCS). MCS refers to a clustering within the same individual of hyperinsulinemia, mild glucose intolerance, dyslipidemia, and hypertension; which are all coronary heart disease (CHD) risk factors that are also associated with PCOS (6-8). In a study which was undertaken in the USA in 2003, the metabolic syndrome was diagnosed in 46% of PCOS women. Another study in the USA confirmed these data (9, 10).

Cystatin C is an extracellular cysteine protease inhibitor that belongs to the cystatin superfamily (11). Cystatin C is produced in all tissues and is present in all biological fluids preferentially in cerebrospinal fluid, seminal plasma and milk. Through regulating cysteine protease activity, it has been reported to be involved in many disease processes, such as inflammation and tumor metastasis (12). 

Cystatin C has also been found to have utility as a biomarker for renal function assessment since it is freely filtered in the renal glomeruli, and completely reabsorbed by the renal tubuli, and cannot be extra-renally eliminated (13-15). Especially, experimental studies suggest that its inhibitory effects on cysteine protease might help keep plaque from destabilization (11). Recent studies have demonstrated that elevated cystatin C serum levels are associated with the risk of cardiovascular events both in asymptomatic elderly subjects and in patients with heart failure or an acute coronary syndrome (16-19).

Some studies support an important diagnostic value of cystatin C among patients with metabolic syndrome. Cystatin is a useful clinical marker that provides additional information to the established risk determinants (20, 21). Thus, cystatin C could be a useful tool in patients with PCOS who are at high risk of metabolic syndrome and cardiovascular disease. We therefore sought to investigate relationship of cystatin C and PCOS. 

## Materials and methods

**Population**

In this case-control study, 35 consecutive women aged between 15 and 35 with PCOS were evaluated. The diagnosis of PCOS was based on NIH criteria (hyperandrogenism and chronic anovulation). Patients were excluded from participation if they were pregnant, or had diabetes, thyroid disease, adrenal disorder, hepatic, cardiovascular disorders, or abnormal renal fuction (GFR<60cc/min). No subjects were treated with corticosteroids, statins, aspirin, oral contraceptives or any other medication for at least 3 months prior to participation in the study. 

Most women in this study had evidence of polycystic ovaries on ultrasound, but this was not noted as a criterion for diagnosis. Clinical hyperandrogenism was described by the presence of hirsutism, acne, or androgenic alopecia. Patients who have scors more than 8 (Ferriman-Gallwey-Lorenzo scores) were defined as hirsute. (22). Biochemical hyperandrogenism was defined as serum androgens levels (testosterone or dehydroepiandrosteronesulphate>2 SD above the reference mean values) elevating (23). 

Other causes of hyperandrogenism, such as hyperprolactinemia, Cushing’s syndrome, and congenital adrenal hyperplasia, were excluded. Anovulation was defined as serum progesterone <2ng/mL. In patients with normal menses, studing atleast 2 consecutive menstrual cycles showed low levels of serum progesterone (<2ng/mL) in both cycles in some of them who suffered from chronic anovulation (24).

35 age and body mass index (BMI) matched normal ovulatory women were also enrolled in the study. The same exclusion criteria were used for the control group as patient group. The research protocol was approved by the Human Research Ethics Committee of Kerman University of Medical Sciences (H-408-0407). All patients and control subjects were voluntarily recruited for the study after being totally, informed about the issue.

**Laboratory assay**

Blood samples were withdrawn by venipuncture between 8:00 and 9:30 AM after an overnight fast and then centrifuged; aliquots of serum were stored at -80^o^C until assayed. Serum levels of cystatin C and CRP were measured by high-sensitive immunonephelometric method (Dade Behring, Marburg, Germany) (25). 

As for CRP the assay detection limits were 0.175 mg/l and inter and intra-individual coefficients of variation were <6% and <4.5%, respectively. As for cystatin C the assay detection limits were 50 ng/ml and the inter- and intra-individual coefficients of variation were <3.6% and <2.7%, respectively. According to previous studies we chose cut-off values for CRP of 3 mg/l (26). 

Routine biochemical measurements, including creatinine, were determined on serum sample using an autoanalyzer. Total cholesterol and triglyceride concentrations were determined by an automated enzymatic method (Bio Merieux, Marcy l’Etoile, France), and high density lipoprotein (HDL) cholesterol by an enzymatic procedure after phosphotungstic acid/ magnesium chloride precipitation of apoB-containing lipoproteins. 

Low-density lipoprotein (LDL) cholesterol concentration was calculated according to the Friedewald formula when triglyceride levels were <400mg/dL. All laboratory measurements were performed in blinded fashion. Height, body weight and blood pressure were measured in each subject. The BMI was calculated according to the following formula: body weight in kilograms/height in m^2^.

**Statistical analysis**

SPSS for Windows Version 16 (Chicago, IL) was used for data management and statistical analysis. Data are expressed as the mean±SD. Because variables of interest were non-normally distributed, nonparametric statistical methods were used to analyze the data. Comparing the PCOS and control groups with respect to non-categorical variables was performed using Mann-Whitney test. Chi-square test of association was used to compare independent groups, and Univariate and multivariate logistic regressions were used to estimate the association of individual components with PCOS. The level of statistical significance was 0.05. No one-sided statistical tests were done.

## Results

Baseline demographic, clinical, and laboratory findings of PCOS patients versus control group are summarized in table I. Patients with PCOS exhibited significantly higher systolic and diastolic blood pressure in comparison to healthy subject. As expected, triglyceride and LDL cholesterol levels were higher and HDL cholesterol levels were lower in PCOS patients as compared with healthy population but, all of them were in normal range. However, plasma total cholesterol and lipoprotein A (LPA) levels were not different in the 2 groups. 82.9% of healthy subjects didn’t have dyslipidemia whilst only 52.8% of patients with PCOS had normal lipid profile as difference which was statistically significant (p=0.007). Cystatin levels were significantly higher in patients with PCOS compared to healthy subjects.

A positive correlation was seen between cystatin C, and PCOS which was statistically significant (r=0.54). Likewise, as cystatin C increases systolic and diastolic blood pressure are increased too; but the correlation with dyslipidemia is not significant. In the same way, no correlation was found for cystatin and LPA, but there was a statistically significant positive relationship between cystatin C and CRP values. All values and significance are shown in table II.

Multiple linear regressions was conducted to estimate the overall prediction of cystatin C predicting factors as a function of PCOS status, age, body mass index, systolic and diastolic blood pressure, lipid profile, CRP and LPA (Table III). Nevertheless, for the overall model, only PCOS status was independently associated with Cystatin C (p<0.0001). 

**Table I:** Comparison of demographic, analytical, clinical, and laboratories characteristics between subjects with and without PCOS

**Variables**	**PCOS (n=35)** **(mean±SD)**	**Healthy volunteers (n=35)** **(mean±SD)**	p-value*
Age (year)	26.3 ± 4.6	25.1 ± 0.7	0.493
Body Mass Index (kg/m^2^)	27.3 ± 4.7	28.5 ± 5.5	0.514
Systolic blood pressure (mmHg)	117.3 ± 9	107.4 ± 9	<0.001
Diastolic blood pressure (mmHg)	76.5 ± 6.8	68.8 ± 6.9	<0.001
Mean arterial pressure (mmHg)	90.1 ± 6.2	81.7 ± 6.8	<0.001
Triglyceride (mg/dL)	93.8 ± 37.3	73.4 ± 27.9	0.012
Cholesterol (mg/dL)	169.5 ± 30.9	157.1 ± 31.9	0.099
HDL (mg/Dl)	50.9 ± 9.5	59.5 ± 9.3	<0.001
LDL (mg/dL)	119.5 ± 22.7	100.1 ± 27.6	0.002
LPA(mg/dL)	23.4 ± 11.1	20.6 ± 9.6	0.282
CRP (µg/ml)	4.1 ± 2.3	2.1 ± 2	<0.001
Cystatin C (ng/ml)	1255 ± 175	1022 ± 183.1	<0.001

HDL: high density lipoprotein          LDL: low density lipoprotein          LPA: lipoprotein A          CRP: C reactive protein

* independent t-test

**Table II:** Correlation between cystatin C and variables

**Variables**	**Cystatin C**	**p-value**
Age	0.111	0.179
Body mass index	0.137	0.123
Systolic blood pressure	0.299	0.011
Diastolic blood pressure	0.313	0.008
Dislipidemia	0.151	0.210
CRP	0.615	<0.001
LPA	-0.022	0.860

* Pearson correlation test.

**Table III:** Multivariate linear regression: cystatin C

**Variables**	**Beta**	**95% CI ** **Lower bound**	**95% CI** **Upper bound**	**p-value**
PCOS	0.45	98.2	290.5	<0.001
Age	0.06	-4.8	10.7	0.457
Body mass index	0.45	-8.9	27.6	0.218
Systolic blood pressure	-0.041	-5.3	3.6	0.702
Diastolic blood pressure	-0.057	-7.6	4.5	0.615
Dyslipidemia	-0.107	-138.3	39.7	0.273
CRP	0.156	-7.1	34.3	0.193
LPA	-0.018	-3.9	3.2	0.835

* Multivariate linear regression test.

## Discussion

In this study, we report an association between cystatin C and PCOS. Likewise, it was associated with both systolic and diastolic blood pressure in a positive way. These correlations, except PCOS status, did not persisted after adjusting other measured variables. Thus, our study suggests that PCOS is an important determinant of cystatin C levels.

Several recent publications have demonstrated that cystatin is superior to serum creatinine or creatinine-based estimating equations for prediction of all-cause mortality, cardiovascular events and incident congestive heart failure in cohorts that were predominantly free of cardiovascular disease at inception (17, 27-30). Furthermore, in our study the possibly important prognostic value of cystatin among patients with PCOS has also been suggested and it might be a useful clinical marker providing complementary information to establish risk determinants. 

PCOS is strongly and independently associated with higher level of cystatin. This suggests that the information obtained from cystatin is not just a marker of glomerular filtration. Servais *et al* showed that cystatin level is significantly related with 3 metabolic syndrome components: elevated blood pressure, increased triglycerides, and increased waist circumference, however, it has only significant correlation with blood pressure and CRP in our result (21). 

Rapul *et al* showed that young people with PCOS without cardiovascular risk factor have subclinical early coronary atherosclerosis. Based on our study result maybe cystatin C is one of the causes of methabolic syndrom, hypertention and cardiovascular complication in PCOS. This relation of cystatin C and this disorder was confirmed before (31). 

In our study, an inflammatory mechanism does not seem to be involved in the association between cystatin C and polycystic ovary syndrome. Indeed, we failed to demonstrate a correlation between cystatin C and CRP levels. Furthermore, cystatin C is associated with the extent of the disease even after adjusting CRP levels in the multivariable model. Since the mechanism in which PCOS increases cystatin C levels is not known, further studies are required to evaluate the pathways involved in these results.
